# Tension orbit secondary to a carious primary molar—A case report

**DOI:** 10.1002/ccr3.3942

**Published:** 2021-02-16

**Authors:** Mehul R. Jaisani, Ashok Dongol, Pradeep Acharya, Anjani Kr Yadav, Chandrakant Pasvan, Siddhartha Rai, Rikta Pande, Bandana Koirala, Shankar Shah, Poonam Lavaju, Sean Laverick

**Affiliations:** ^1^ Department of Oral and Maxillofacial Surgery B.P Koirala Institute of Health Sciences Dharan Nepal; ^2^ Department of Pedodontics and Preventive Dentistry B.P Koirala Institute of Health Sciences Dharan Nepal; ^3^ Department of Otolaryngology, Head and Neck Surgery B.P Koirala Institute of Health Sciences Dharan Nepal; ^4^ Department of Opthalmology B.P Koirala Institute of Health Sciences Dharan Nepal; ^5^ Department of Oral and Maxillofacial Surgery Ninewells Hospital NHS Tayside Dundee UK; ^6^ BPKIHS Dharan Nepal

**Keywords:** orbital cellulitis, primary molar, tension orbit, vision

## Abstract

Though rare, a pediatric dentist should have background knowledge of this kind of presentation which can greatly affect their patient's quality of life and leave them with a significant deficit at a very young age.

## Graphical abstract

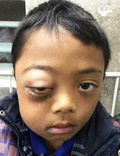

## 1 INTRODUCTION

Tension orbit is a clinical condition resulting from intraorbital space compression, manifesting as severe proptosis leading to stretching of the optic nerve and threatening vision. Tension orbit can be secondary to orbital cellulitis. Though rare but orbital cellulitis can be the sequale of odontogenic infection. In this case report, we present a case of an 8 years old boy who presented with progressive orbital cellulitis causing endangered vision secondary to carious primary maxillary molar. The patient was appropriately managed with intravenous antibiotics and surgical decompression. Significant morbidity was overcome by multi‐specialty team approach.

Tension orbit is a clinical condition resulting from intraorbital space compression, manifesting as severe proptosis leading to stretching of the optic nerve and threatening vision. Tension orbit can be secondary to orbital cellulitis. The most common predisposing factor for orbital cellulitis is sinus disease, particularly in the younger age group.^1^ Orbital cellulitis can also arise from odontogenic causes, even though its prevalence is rather infrequent, comprising only 2%‐5% of cases.^2^ Though rare, acute periapical infections causing periorbital cellulitis can lead to significant morbidity; such as impaired or loss of vision, cavernous sinus thrombosis and even brain abscesses, the latter two having a high mortality rate when untreated.

## 2 CASE REPORT

**FIGURE 1:**
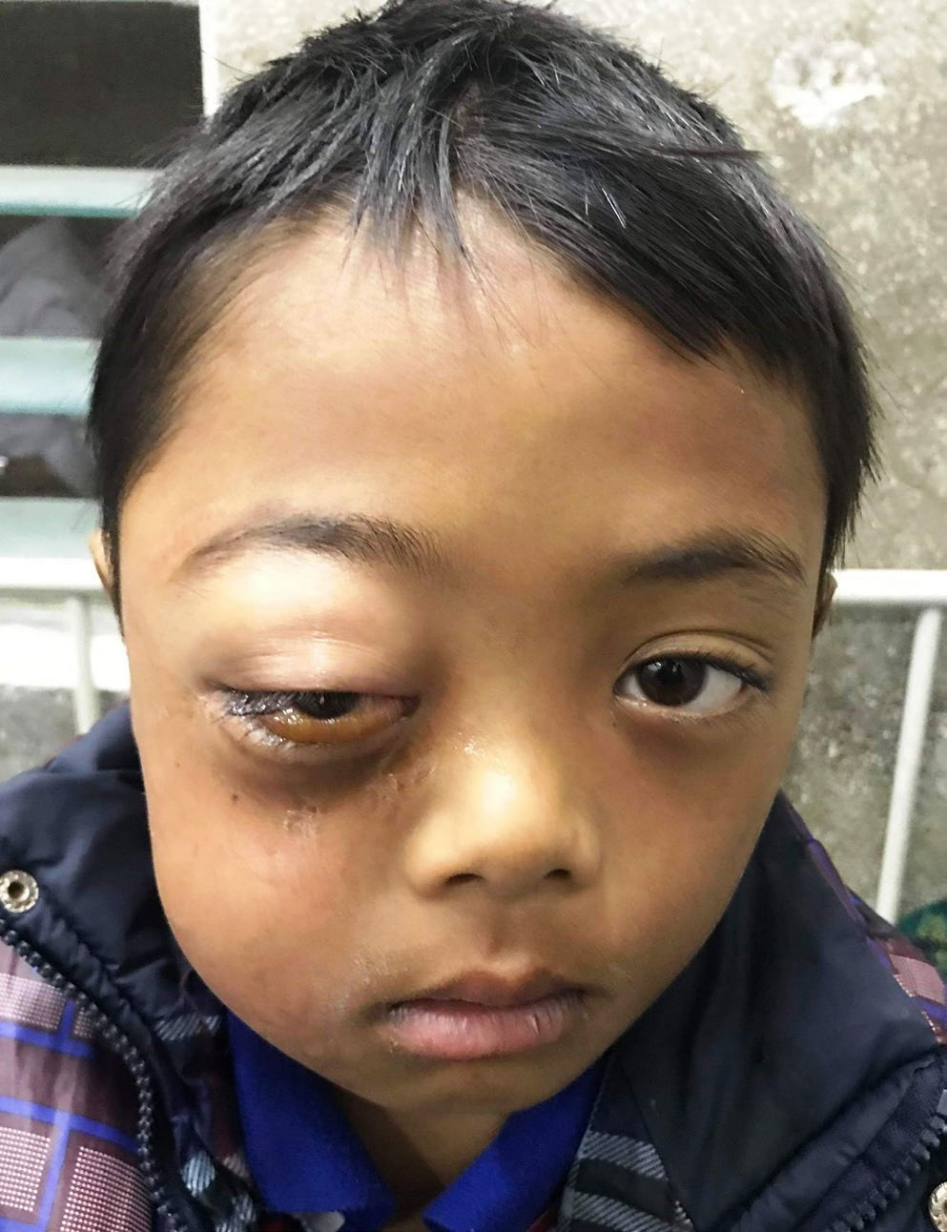
Proptosis, chemosis, and right cheek swelling at initial presentation

**FIGURE 2:**
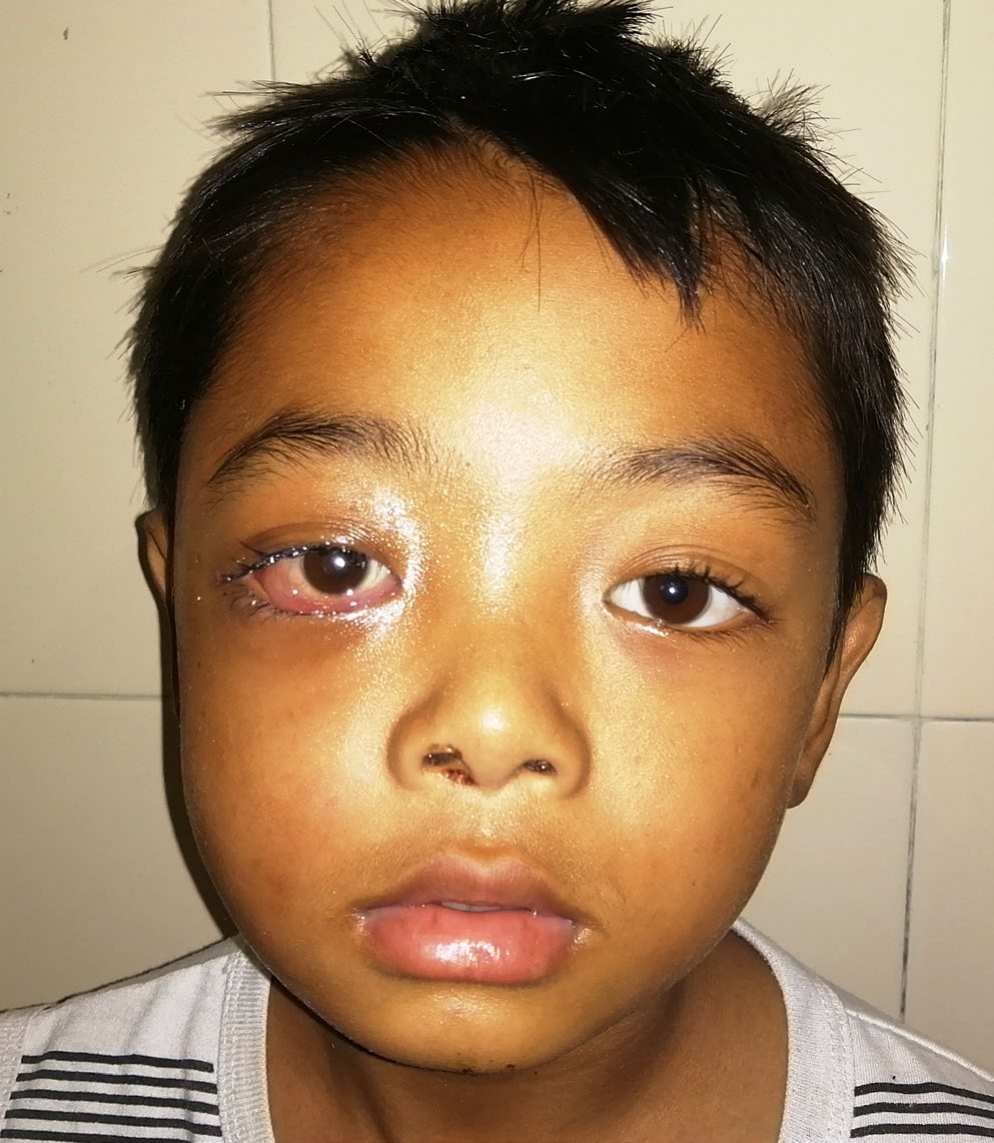
Residual chemosis and mild proptosis at the time of discharge

**FIGURE 3:**
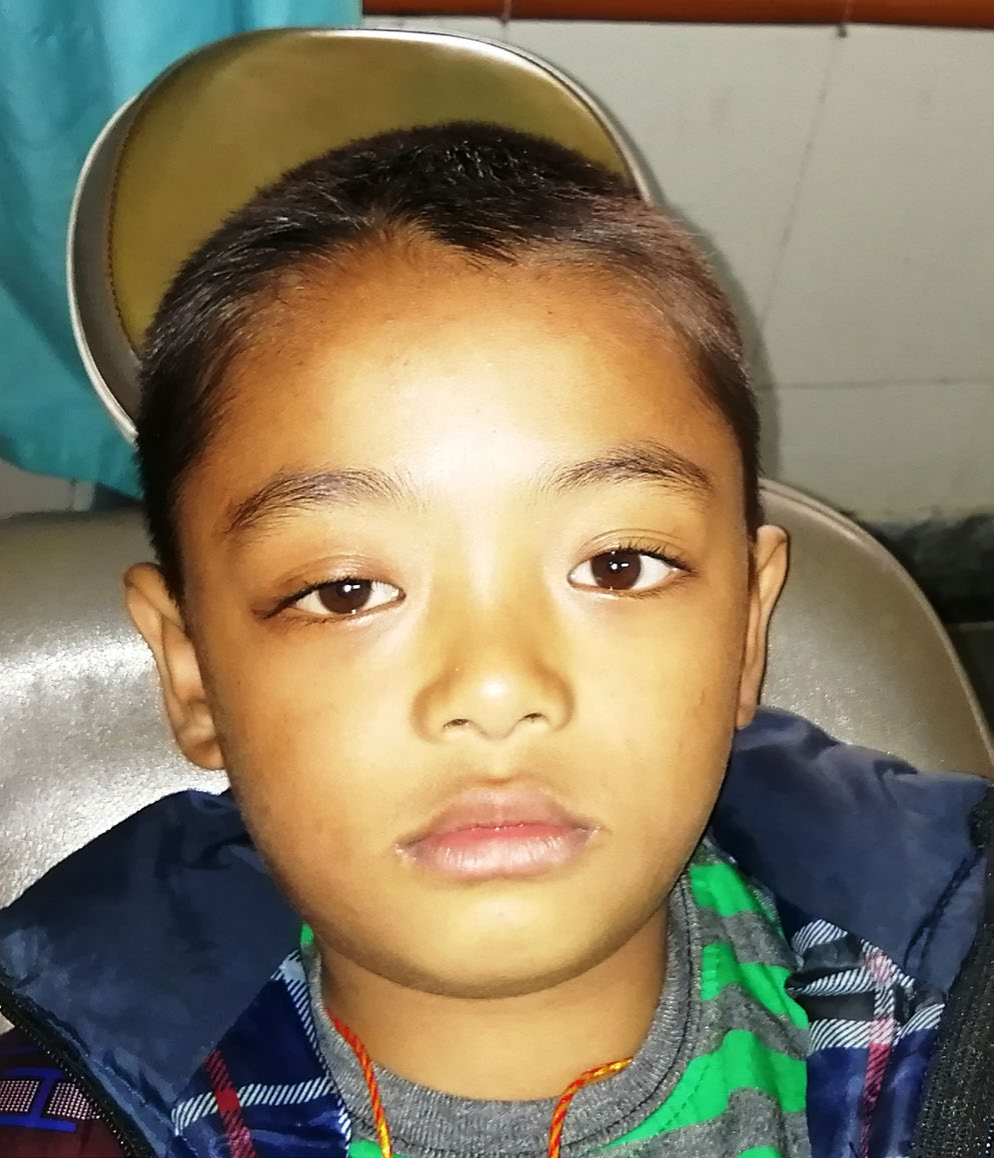
At 1 mo follow‐up

An 8‐year‐old boy presented to the emergency department with a 4 day history of swelling of the right periorbital region. He also reported having tooth ache in the right maxilla for the preceding week, which was followed by fever. At presentation, he had a fever, leucocytosis, swelling over the right cheek and periorbital region with a proptosed eye. Inter‐incisal mouth opening was approximately 7 mm, limiting intraoral examination. There was marked vestibular obliteration with a carious right upper deciduous second molar. Ophthalmological examination revealed right eye axial proptosis, diffuse swelling over ispsilateral eyelids, diffuse chemosis, mild limitation in extraocular movements, ophthalmoplegia, with a normal fundus (Figure 1). A diagnosis of buccal and periorbital facial space infection secondary to carious 55 with periorbital cellulitis was made. The patient was started on intravenous antibiotics, steroids, and planned for surgical decompression under general anesthesia (GA). Extraction of 55 and intraoral surgical drainage of right side buccal and infraorbital space was performed. Despite marked improvement in inter‐incisal mouth opening and reduction in facial swelling, ophthalmologic findings deteriorated from third postoperative day. The patient developed lagophthalmos with diffuse congestion and pus within the conjunctiva with spikes within the cornea. Fundal examination revealed a hyperemic disk with striations around the macula. An emergency contrast enhanced CT showed fluid collection within the right maxillary, ethmoid and sphenoid sinuses with similar collections in the lower and outer quadrants of the extraconal space and intraconal space within the orbit, displacing inflamed extraocular muscles and the globe anteriorly. A lateral canthotomy and functional endoscopic sinus surgery was done under GA to drain the sinuses. The patient showed marked improvement postintervention, with return of the fundus and cornea to normal. However, there was still some axial proptosis, congestion and chemosis of the right eye on discharge (Figure 2). The patient was discharged with instructions for eye care. One month later, the patient completely recovered without any residual ophthalmological complications and returned to normal appearance (Figure 3).

## 3 DISCUSSION

Orbital cellulitis secondary to odontogenic sources is a relatively rare complication. The route of spread can be explained on the basis of the anatomic relations of the facial bones. The most common pathway is via the sinus because the roots of molar and premolar tooth are adjacent to the base of maxillary sinus. The inflammation or infection of the sinus can then spread into the orbit through bone erosion between the orbit and the maxillary sinus or through the ethmoid sinus or the infraorbital canals.^3^ Alternatively, it can spread through the facial soft tissues over the buccal cortical plate, spreading to periorbital tissues. Thirdly, infection of a molar or premolar tooth invades the infratemporal and pterygopalatine fossa, spreading into the orbit through the inferior orbital fissure.^3^, ^4^, ^5^ Infection of a tooth can also spread into the orbit along the facial vein and the ophthalmic vein by hematogenous regurgitation because the veins of the face, eyes, nasal cavity, and sinuses are all connected without valves.^3^ The finding of sinusitis and facial space infection from a carious deciduous molar in our patient supports the first two possible routes of spread. Proptosis, eyelid swelling, conjunctival chemosis, and limited ocular mobility are features suggesting orbital cellulitis. The specific worrying features are decreased visual acuity, proptosis, and external ophthalmoplegia. A temperature greater than 37.5°C and leukocytosis is a more prominent feature in the pediatric group. As noted in a literature review,^5^ 75.7% have an identifiable dental lesion or symptoms compatible with an oral inflammatory process.

A CT‐scan is indicated in all patients with periorbital inflammation having proptosis, ophthalmoplegia, or decreased visual acuity, also in cases where a foreign body or an abscess is suspected, severe eyelid edema preventing adequate examination, or in whom surgery is contemplated.^1^

Early empirical use of intravenous broad‐spectrum antibiotics should be administered. Antibiotics are backed‐up by surgical intervention for significant underlying sinus disease, orbital or sub‐periosteal abscess. Functional endoscopic sinus surgery (FESS) is preferable over conventionally used periorbital incisions.

## 4 CONCLUSION

- Carious primary teeth when neglected is enough to cause mortality such as loosing vision and death.
- Multidisciplinary team approach can overcome a significant morbidity and mortality and greatly affect the quality of life.

## CONFLICT OF INTEREST

None.

## AUTHOR CONTRIBUTIONS

MRJ: managed the patients and wrote the manuscript. AD, PA, AY, BK, SS, and PL: managed the patients, involved in follow‐up and proof reading. CP, SR, RP: collected the data and managed the patients. SL: involved in manuscript writing and editing.

## ETHICAL APPROVAL

As per Declarations of Helsinki.

## Data Availability

https://doi.org/10.22541/au.161065148.85180129/v1
